# Low prevalence of relevant findings in inappropriate echocardiograms and discordant perceptions between cardiologists and patients

**DOI:** 10.1590/1414-431X20187413

**Published:** 2018-05-28

**Authors:** J.R. Lopes, A.C. Oliveira, V.G. Rios, L.C.L. Correia

**Affiliations:** 1 Escola Bahiana de Medicina e Saúde Pública, Brotas, Salvador, BA, Brasil; 2 Cardioclin, Conceição do Coité, BA, Brasil; 3 Hospital São Rafael, Salvador, BA, Brasil

**Keywords:** Echocardiogram, Inappropriate, Utility, Patients, Cardiologists

## Abstract

Complementary examinations are “inadequate” whenever the likelihood of benefits from their indication is lower than the negative results. The low benefit is a result of poor performance in detecting relevant changes that lead to improved behavior. However, inadequate examinations are prevalent and little is known about patients' notions of the usefulness of such indications. The aim of this study was to describe relevant findings in inappropriate echocardiograms and to assess the level of agreement between patients and cardiologists regarding their usefulness. Adults without known cardiovascular disease who were referred for echocardiogram by inappropriate criteria according to the American College of Cardiology were selected. Relevant findings were defined by any change in the degree of moderate to severe, according to the American Society of Echocardiography. We tested the level of agreement between the patients who underwent echocardiographic examination and the physicians who requested the exam through a standard questionnaire. Five hundred patients were included, with average age of 52±17 years (47% males). Only 17 patients had any relevant changes (3.4%, 95%CI=2 to 5.4%). The most frequent alterations included valve changes in 8 and diastolic dysfunction grade II in 6 patients. Eighty-seven examinations were performed to determine the level of agreement between patients and cardiologists. For the question “Is this test really necessary?”, 92% of patients responded positively, compared with 5% of cardiologists (Kappa negative 0.04; P=0.01). The frequency of relevant findings was low in inadequate echocardiograms and patients and cardiologists had a different perception regarding its usefulness.

## Introduction

Echocardiography is an imaging method that allows for the diagnosis and management of cardiovascular diseases (1,2), representing approximately half of the diagnostic tests performed in cardiac imaging services (3). In many cases, it is used as a screening tool in patients with low cardiovascular risk (4).

Some studies showed a marked increase in the number of echocardiogram requests in recent years (6–8% per year) (3,5). In Brazil (2010), 715,655 elective echocardiograms were performed in outpatients in the Unified Health System (SUS), and 425.317 (59%) of them were performed in the southeastern region of the country, with an estimated cost of over R$ 30 million (6), contributing to a progressive increase of public health costs (7).

To improve clinical practice, reduce unnecessary exams, and optimize the cost-effectiveness relationship, the American College of Cardiology and the American Society of Echocardiography published the Appropriate Use Criteria for requesting echocardiograms (8). Different clinical scenarios were classified as appropriate, uncertain or inappropriate according to a score system based on the reason for the request. Inappropriate exam requests are those with inadequate indication and that will likely not provide benefit to the patient. Since then, some actions have been taken to try to improve this scenario and reduce the number of inappropriate echocardiograms, and the Choosing Wisely campaign was launched (9,10).

In some articles, the varied prevalence of important findings in inappropriate echocardiograms may reflect the study population (11–14). Barbosa et al. (15) did not find a difference between public and private hospitals in the adequacy of transthoracic echocardiogram requests for outpatients.

Therefore, the objectives of this study were to i) describe the frequency of relevant findings in echocardiograms with inappropriate indication and ii) to evaluate the agreement between physicians and patients regarding their perception of the utility of the echocardiographic tests performed.

## Material and Methods

### Sample selection

From September 2016 until April 2017, all patients who underwent echocardiograms in a private office in the city of Conceição do Coité (Brazil) were screened for the study. Inclusion criteria were defined as follows: age>18 years, absence of previously diagnosed heart disease, echocardiogram considered inappropriate, acceptance to participate in the study, and signature of the free and informed consent form.

Prior heart disease was defined based on the known diagnosis of acute or chronic heart disease, characterized by structural change in morphology and/or reduction in functional heart capacity. Inadequate echocardiogram was defined according to the appropriate use criteria of the American Society of Echocardiography (Appropriate Use Criteria for Echocardiography, 2011) (8). The concept of appropriateness establishes that an “appropriate imaging test is one in which the incremental information combined with clinical judgment exceeds the possible negative consequences for a large margin of specific indications, in which the procedure is considered acceptable and reasonable”. The request indications are classified as appropriate, uncertain, and inappropriate, according to a score system (9–7, 6–4, and 3–1, respectively) (8). Recently, a new nomenclature was proposed: an appropriate test would be called “test with appropriate care”, an uncertain test would be called “test with possible appropriate care”, and inappropriate test would be called “test with rarely appropriate care” (9).

### Study protocol

Patients arriving at the office to undergo echocardiography were individually informed about the study and invited to participate. Study details were explained, and patients received the free and informed consent form to read; they were included in the study only after their formal written consent.

Data collection was performed as an interview in an office environment, with the completion of a structured data collection sheet preferably by the patients or with the assistance of the investigator or an accompanying person in case the patient was illiterate. The information was related to patient identification, anamnesis, details of symptoms, and elucidation of the reason for the test request (with the aid of the request guide signed by the attending physician when available). A question was made regarding the expectation of the patient and cardiologist on the utility of the echocardiogram result. Numerical identification codes for the patient and requesting physician were created to protect the names of research participants. As the service cardiologists were also interviewed, they also received the free and informed consent form.

After the interview, each patient was referred to another office to undergo the test. The physician who performed the echocardiogram did not participate in the study and, therefore, did not have access to the patients' data sheet. Next, two copies of the test were printed, one given to the patient to be sent to the physician, and the other attached to the patient's file. Then, the investigator started the analysis of each exam to classify its adequacy using the list with the 98 indications for the transthoracic echocardiogram, according to the Appropriate Use Criteria for Echocardiography (2011) (8).

### Definition of relevant findings

The echocardiographic findings showing changes compatible with structural or functional heart diseases were defined as relevant in moderate to severe degrees according to the recommendations of the American Society of Echocardiography (1,16). The findings considered relevant in inappropriate echocardiograms are as follows: 1) systolic dysfunction of the left ventricle (Simpson) when the ejection fraction is <40%; 2) diastolic dysfunction of the left ventricle (grades II-IV); 3) change in the left ventricular contractility (hypokinesia, akinesia, or dyskinesia); 4) valvar changes (at least moderate), mitral stenosis (valvar area <1.5 cm^2^); mitral insufficiency with vena contracta value >3 mm and/or jet/AE area ratio >20%; aortic stenosis with mean gradient >30 mmHg or maximum gradient >50 mmHg; aortic insufficiency when the ratio between the regurgitant jet width/VSVE >25% or the regurgitant jet length >2 cm of the aortic valve is demonstrated; tricuspid insufficiency as expressed in the ratio between jet volume/right atrium >50% and/or vena contracta width >1 cm; 5) dilation of the left ventricle: men (diastolic diameter >64 mm and systolic diameter >44 mm) and women (diastolic diameter >57 mm and systolic diameter >39 mm); 6) dilation of the right ventricle: (baseline diameter >41 mm and/or mean diameter >35 mm); 7) pulmonary arterial hypertension when the systolic pressure of the pulmonary artery is >45 mmHg; 8) cardiac masses or tumors: (*eg*, atrial myxomas, vegetations, and intracavitary thrombi); 9) changes in the pericardium, pericardial fluid involving the whole heart, and signs of pericarditis with thickening of >2 mm; 10) congenital heart disease: (*eg*, interatrial communication, ventricular septal defect, aortic stenosis, pulmonary stenosis, and subaortic stenosis).

### Perception of cardiologists and patients

Comparison between proportions of questions asked to physicians and patients was evaluated in 97 tests, since only service cardiologists participated in this phase of the study. Three equal questions were asked to patients and cardiologists. The first question (“In your opinion, this test is”) had two possible answers: “little necessary” or “very necessary”. The second question (“What is the likelihood of this test to detect an important change?”) also had two possible answers: “low likelihood” or “high likelihood”. The third question (“You are undergoing this test”) had three possible answers: “for checkup (no symptoms)”, “to investigate the cause of symptoms”, or “to evaluate existing disease”.

### Statistical analysis

To evaluate the frequency of relevant findings in inappropriate echocardiograms (first objective), the study sample size was calculated by estimating a 5% prevalence of useful tests to obtain a 95%CI (range: ±2%). Thus, 460 inappropriate echocardiograms were required.

Regarding the analysis of agreement between cardiologists and patients on their perceptions of the utility of inappropriate echocardiograms (second objective) and given the final number (97) of tests requested by staff cardiologists, a sample size was calculated *a posteriori* (Kappa=0.67) to achieve a statistical power of 80% (alpha=5%).

Categorical variables were reported as either frequencies or percentages. The chi-squared test was used to compare the inappropriate test requests with sample characteristics. P values <0.05 were considered significant for all statistical tests. All statistical analyses were performed using the Statistical Package for the Social Sciences (SPSS, v.17, USA) software.

The tests were performed by a single medical echocardiographist who used an Echocardiography Phillips HD7¯ (Bothell, USA) equipment. The study project was approved by the Research Ethics Committee of the Escola Bahiana de Medicina e Saúde Pública (No. 1,661,798; CAAE # 55513416.8.0000.5544). The study was conducted in accordance with the National Health Council (CNS) resolution (# 466/2012), meeting the guidelines and norms regulating research involving human beings.

## Results

### Sample characteristics

From September 2016 to April 2017, 1075 echocardiogram tests were performed in this cardiology service; however, four of these patients refused to participate in the study. A total of 1071 patients signed the free and informed consent form; 569 of them had their tests classified as inappropriate (53%), and 69 of these were excluded because heart disease was previously diagnosed. Thus, the study sample consisted of 500 patients (age: 52±17 years), and their distribution between men and women was balanced. No test was covered by SUS; about half (51%) of the tests were covered by private health plans and the remainder were paid by the patients. In this group, 11% were illiterate and only 26% had university education (Table 1).

**Table 1.** Baseline characteristics of the study population.

Clinical characteristics	Frequencies (%)
Sample	500
Age (years, mean±SD)	52±17
Males	236 (47)
Health plan	255 (51)
Patients education	
Illiterate	53 (11)
Primary school	113 (24)
High school	184 (39)
University	126 (26)
Cardiovascular symptoms	197 (39)
Dizziness	73 (15)
Palpitations	67 (13)
Dyspnea	57 (11)
Chest pain	37 (7)
Edema in lower limbs	13 (3)
Syncope	7 (1)
Presence of chronic disease or clinical change	256 (51)
Systemic arterial hypertension	224 (45)
Dyslipidemia	67 (13)
Preoperative	47 (9)
Diabetes mellitus	38 (8)
Heart murmur	4 (1)
Renal insufficiency	3 (0.6)
Systemic arterial hypertension	224 (45)
Dyslipidemia	67 (13)
Preoperative	47 (9)
Diabetes mellitus	38 (8)
Heart murmur	4 (1)
Renal insufficiency	3 (0.6)
Physical activity	195 (39)
Alcoholism	53 (11)
Smoking	24 (5)
Medications used	
At least one of the medicines	219 (44)
Angiotensin receptor blocker	121 (24)
Diuretic	69 (14)
Beta blocker	65 (13)
Calcium channel blocker	35 (7)
Angiotensin Converting Enzyme Inhibitor	25 (5)
Reasons for requesting the echocardiogram	
Routine assessment of systemic hypertension without symptoms or signs of HHD	204 (40.8)
Initial evaluation of ventricular function (screening)	185 (37)
Peri-operative routine evaluation	47 (9.4)
Assessment of dizziness or pre-syncope	31 (6.2)
Assessment of premature atrial contraction	19 (3.8)
Evaluation of ventricular function with normal pre-evaluation	8 (1.6)
Evaluation of asymptomatic sinus bradycardia	3 (0.6)
Routine assessment of vestigial valve regurgitation	2 (0.4)
Initial assessment with no sign or symptom of structural heart disease	1 (0.2)

HHD: hypertensive heart disease.

Most patients were asymptomatic and 39% had symptoms, including dyspnea, chest pain, palpitation, edema in the lower limbs, dizziness, and syncope. The percentage of patients with symptoms related to the cardiovascular system was significant. However, their conditions did not imply a significant likelihood of heart disease, not meeting the criteria for appropriate use of the test.

Half of the patients presented some type of morbidity, and systemic arterial hypertension was the most prevalent. However, cases of disabling disease were not observed (39% of patients had regular physical activity). A total of 44% of patients had regular use of medications, most of which were antihypertensive drugs (Table 1).

### Relevant echocardiographic findings

Only 17 (3.4%) patients presented some relevant change on the echocardiogram (95%CI=2.0–5.4%). The most frequent changes were moderate valve changes (8 patients), followed by diastolic dysfunction grade II (6 patients), and moderate (two patients) and marked (two patients) left ventricular systolic dysfunctions. Findings corresponding to pulmonary arterial hypertension, dilation of the right ventricle, cardiac masses or tumors as well as pericardial changes were not observed in the tests (Table 2).

**Table 2.** Proportion of relevant findings in inappropriate echocardiograms.

Relevant findings	Frequencies (%)	95%CI
Tests with at least one relevant finding	17 (3.4)	2.0–5.4
Systolic dysfunction		
Moderate	2 (0.4)	0.05–1.4
Important	2 (0.4)	0.05–1.4
Diastolic dysfunction		
Grade II	6 (1.2)	0.44–2.6
Grade III	0	
Grade IV	0	
Change in contractility		
Hypokinesia	1 (0.2)	0.01–1.1
Akinesia	2 (0.4)	0.05–1.4
Dyskinesia	0	
Valvar change	8 (1.6)	0.7–3.1
Moderate mitral insufficiency	4 (0.8)	0.22–2.6
Important mitral insufficiency	1 (0.2)	0.01–1.1
Moderate aortic stenosis	1 (0.2)	0.01–1.1
Moderate aortic insufficiency	1 (0.2)	0.01–1.1
Important aortic insufficiency	1 (0.2)	0.01–1.1
Dilatation of the left ventricle		
Moderate	0	
Important	2 (0.4)	0.05–1.4
Dilatation of the right ventricle		
Moderate	0	
Important	0	
Pulmonary arterial hypertension	0	
Congenial heart disease		
Subvalvar aortic stenosis	1 (0.2)	0.01–1.1
Cardiac masses or tumors	0	

Relevant echocardiographic findings were observed only in three types of indications for inappropriate echocardiogram request. Most of these findings occurred in tests whose indication corresponded to a routine evaluation of systemic arterial hypertension (with no sign or symptom of hypertensive heart disease; 13 tests), followed by routine perioperative evaluation of ventricular function (with no sign or symptom of cardiovascular disease; 3 tests), and indication of initial evaluation of ventricular function (screening; with no sign or symptom of cardiovascular disease; 1 test).

### Agreement in perception by cardiologists and patients

Most patients (92%) and few cardiologists (5%) responded that the test was very necessary, with agreement of only 9% (negative Kappa of 0.04; statistically significant for disagreement; P=0.01). Most patients (90%) and few cardiologists (6%) answered that there was a great chance of finding a significant change, with agreement of only 11% (negative kappa of 0.04; statistically significant for disagreement; P=0.021). Regarding the question about the reason for undergoing the test, 65% of patients and 42% of cardiologists answered “checkup (no symptom)”; whereas 31% of patients and 18% of cardiologists answered “to investigate the cause of symptoms”; and 4% of patients and 40% of cardiologists answered “evaluation of an existing disease” (agreement: 51%; Kappa=0.24; P<0.001; Table 3).

**Table 3.** Agreement between physicians' and patients' perceptions regarding the utility and reason of tests.

Questions	Patients (%)	Physicians (%)	Kappa values	P values
1. Test was much needed	92	5	−0.04	0.01
2. High chance of identifying a significant cardiac change	90	6	−0.04	0.01
3. Reason for the test				
Checkup (no symptoms)	65	42		
Investigation of the cause of symptoms	31	18	0.24	<0.001
Evaluation of an existing disease	4	40		

## Discussion

In the present study, two observations were evident: first, the frequency of relevant findings obtained in echocardiograms requested in inappropriate situations was very low, reinforcing the little utility of these tests; second, there is no agreement between the opinions of cardiologists and their patients regarding utility of these tests, suggesting that the physicians' notion of test utility is not adequately communicated to their patients.

The characteristics of the study population and the situations in which the tests were requested justify the above result. These tests were performed on an elective basis in patients free from previously diagnosed heart disease. In addition, a significant number of patients had initial ventricular function evaluation (screening) as indication, with no sign or symptom of cardiovascular disease. This information shows that echocardiographic tests, which are classified as inappropriate, contribute little to discover important cardiac changes. The impact of these results is likely to be small in relation to future clinical decisions that imply changes in medical conduct regarding patient care. The reason is that the rate of change in the clinical management of patients is considered low even after tests classified as appropriate (17). Therefore, our data reinforce their classification as inappropriate as proposed by the American College of Cardiology.

Given the potential for unintended consequences, efforts should be made to reduce the number of inappropriate echocardiograms such educational interventions (18,19), consulting the appropriateness criteria, use of authorized protocols, and auditing laboratories with evaluation of the echocardiographic requests (20). Recently, Bhatia et al. of the Echo WISELY Trial published an investigator-blind randomized clinical trial, showing the efficacy of educational intervention to reduce inappropriate echocardiograms in outpatients (21). The ultimate goal of these educational interventions has been to benefit patients, as negative consequences are prevented, and reduce health costs.

The proportions of echocardiographic findings considered important obtained in our study was lower than that found in the study published by Koshy et al. (11); these authors found that the prevalence of findings considered abnormal (51%) in tests classified as appropriate was higher than in those classified as inappropriate (38%) (P=0.013). A study by Ward et al. (12) evaluated echocardiograms performed at the outpatient clinic of a university hospital, where new and important abnormalities in appropriate tests (40%) were more common than in inappropriate tests (17%) (P<0.001). Kirkpatrick et al. (13) reported a 20% rate for clinically important new or unexpected echocardiographic findings in tests classified as inappropriate. Similar results were published by Ward et al. (14), who observed a high frequency of new and important abnormalities on echocardiogram tests (classified as inappropriate) carried out in academic centers (16%) and community care centers (15%). In the Tromsø study, in which patients were submitted to echocardiography as screening, a low prevalence of abnormal cardiac findings (7.6%) was observed, and valve disease was the most common finding (22).

However, it is important to emphasize that patients with known diagnosis of heart disease were included in all the studies cited above, differently from our study. The clinical conditions of these patients were also different because patients who were hospitalized or in situations of acute or decompensated illnesses were also included in the other studies. This contributed to the difference in the proportion of echocardiographic findings in relation to our study, which is the first Brazilian evaluation of the frequency of findings considered relevant; in this study, patients without the diagnosis of heart disease submitted to elective echocardiograms classified as inappropriate were included.

Some issues should be mentioned on the disagreement between the views of cardiologists and their patients regarding the clinical utility of the tests and reason to request them. Given the current scenario of medical practice, in which little dialogue occurs between doctors and patients, patients are not sufficiently informed about the consequences of undergoing medical tests. In the study published by Barros et al. (23) on empathy in the doctor-patient relationship in the context of public and private health, low agreement was found in the perception of medical empathy in the public (22%) and private (34.3%) sectors. This suggests that a fragile relationship with little dialogue is established during medical care. Initiatives such as the Choosing Wisely strategy (10) are an advancement, as they try to counteract this scenario by broadening the understanding on inherent aspects of earning interest in the medical practice, allowing the patient to act in decisions related to their health. In addition, analyses have identified situations or factors that may contribute to the excessive use of tests in cardiology services, as cultural reasons also influence these requests. These factors can be classified into clinical or financial, and the reasons may be legal or juridical (24). This is also the first Brazilian study evaluating the agreement between cardiologists and patients regarding inappropriate echocardiograms.

The main limitation of this study is its low representativeness, as it was obtained from an outpatient clinic (convenient sample). Therefore, the applicability of our findings is restricted to populations with characteristics similar to those of our sample. Regarding the issue of doctor-patient communication, our findings may raise the possibility that patients are unaware of the utility of inappropriate tests.

In conclusion, the present study showed that elective echocardiograms requested in inappropriate situations and performed in patients without a diagnosis of heart disease present a low frequency of relevant findings. In addition, patients and cardiologists may have very different perceptions regarding utility of inappropriate tests.

## References

[1] Cheitlin MD, Alpert JS, Armstrong WF, Aurigemma GP, Beller GA, Bierman FZ (1997). ACC/AHA guidelines for the clinical application of
echocardiography: executive summary. A report of the American College of
Cardiology/American Heart Association Task Force on practice guidelines
(Committee on Clinical Application of Echocardiography). Developed in
collaboration with the American Society of Echocardiography. J Am Coll Cardiol.

[2] Garbi M, McDonagh T, Cosyns B, Bucciarelli-Ducci C, Edvardsen T, Kitsiou A (2015). Appropriateness criteria for cardiovascular imaging use in heart
failure: report of literature review. Eur Heart J Cardiovasc Imaging.

[3] Pearlman AS, Ryan T, Picard MH, Douglas PS (2007). Evolving trends in the use of echocardiography: a study of
medicare beneficiaries. J Am Coll Cardiol.

[4] Colla CH, Sequist TD, Rosenthal MB, Schpero WL, Gottlieb DJ, Morden NE (2015). Use of non-indicated cardiac testing in low-risk patients:
Choosing Wisely. BMJ Qual Saf.

[5] Blecker S, Bhatia RS, You JJ, Lee DS, Alter DA, Wang JT (2013). Temporal trends in the utilization of echocardiography in
Ontario, 2001 to 2009. JACC Cardiovasc Imaging.

[6] Barachi LB, Barbosa FC, Moscavitch SD, Pilon BC, Pissinati GV, Mesquita ET (2012). Inadequate request of transthoracic echocardiography according to
the guidelines of the Brazilian Society of Cardiology. Arq Bras Cardiol.

[7] Douglas PS (2008). Quality in echocardiography: choosing to succeed. J Am Soc Echocardiogr.

[8] ACCF/ASE/AHA/ASNC/HFSA/HRS/SCAI/SCCM/SCCT/SCMR (2011). Appropriate Use Criteria for Echocardiography. A Report of the
American College of Cardiology Foundation Appropriate Use Criteria Task
Force, American Society of Echocardiography, American Heart Association,
American Society of Nuclear Cardiology, Heart Failure Society of America,
Heart Rhythm Society, Society for Cardiovascular Angiography and
Interventions, Society of Critical Care Medicine, Society of Cardiovascular
Computed Tomography, and Society for Cardiovascular Magnetic Resonance
Endorsed by the American College of Chest Physicians. J Am Coll Cardiol.

[9] Bhatia RS, Alabousi M, Dudzinski DM, Weiner RB (2016). Appropriate use criteria: a review of need, development and
applications. Expert Rev Cardiovasc Ther.

[10] Bhatia RS, Ivers N, Yin CX, Myers D, Nesbitt G, Edwards J (2015). Design and methods of the Echo Wisely (Will Inappropriate
Scenarios for Echocardiography Lessen Significantly) study: An
investigator-blinded randomized controlled trial of education and feedback
intervention to reduce inappropriate echocardiograms. Am Heart J.

[11] Koshy TP, Rohatgi A, Das SR, Price AL, deLuna A, Reimold N (2015). The association of abnormal findings on transthoracic
echocardiography with 2011 appropriate use criteria and clinical
impact. Int J Cardiovasc Imaging.

[12] Ward RP, Mansour IN, Lemieux N, Gera N, Mehta R, Lang RM (2008). Prospective evaluation of the clinical application of the
American College of Cardiology Foundation/American Society of
Echocardiography Appropriateness Criteria for transthoracic
echocardiography. JACC Cardiovasc Imaging.

[13] Kirkpatrick JN, Ky B, Rahmouni HW, Chirinos JA, Farmer SA, Fields AV (2009). Application of appropriateness criteria in outpatient
transthoracic echocardiography. J Am Soc Echocardiogr.

[14] Ward RP, Krauss D, Mansour IN, Lemieux N, Gera N, Lang RM (2009). Comparison of the clinical application of the American College of
Cardiology Foundation/American Society of Echocardiography Appropriateness
Criteria for outpatient transthoracic echocardiography in academic and
community practice settings. J Am Soc Echocardiogr.

[15] Barbosa FC, Mesquita ET, Barachi LB, Salgado A, Kazuo R, Rosa ML (2011). Comparison of echocardiography request appropriateness between
public and private hospitals. Arq Bras Cardiol.

[16] Lang RM, Badano LP, Mor-Avi V, Afilalo J, Armstrong A, Ernande L (2015). Recommendations for cardiac chamber quantification by
echocardiography in adults: an update from the American Society of
Echocardiography and the European Association of Cardiovascular
Imaging. J Am Soc Echocardiogr.

[17] Matulevicius SA, Rohatgi A, Das SR, Price AL, DeLuna A, Reimold SC (2013). Appropriate use and clinical impact of transthoracic
echocardiography. JAMA Intern Med.

[18] Bhatia RS, Milford CE, Picard MH, Weiner RB (2013). An educational intervention reduces the rate of inappropriate
echocardiograms on an inpatient medical service. JACC Cardiovasc Imaging.

[19] Bhatia RS, Dudzinski DM, Malhotra R, Milford CE, Yoerger Sanborn DM, Picard MH (2014). Educational intervention to reduce outpatient inappropriate
echocardiograms: a randomized control trial. JACC Cardiovasc Imaging.

[20] Fonseca R, Marwick TH (2014). How I do it: judging appropriateness for TTE and
TEE. Cardiovasc Ultrasound.

[21] Bhatia RS, Ivers NM, Yin XC, Myers D, Nesbitt GC, Edwards J (2017). Improving the appropriate use of transthoracic echocardiography:
The Echo WISELY Trial. J Am Coll Cardiol.

[22] Lindekleiv H, Lochen ML, Mathiesen EB, Njolstad I, Wilsgaard T, Schirmer H (2013). Echocardiographic screening of the general population and
long-term survival: a randomized clinical study. JAMA Intern Med.

[23] Barros PS, Falcone EM, Pinho VD (2011). Avaliação da empatia médica na percepção de médicos e pacientes
em contextos público e privado de saúde. Arq Ciênc Saúde.

[24] Huang X, Rosenthal MB (2015). Overuse of cardiovascular services: evidence, causes, and
opportunities for reform. Circulation.

